# Pre-processing by data augmentation for improved ellipse fitting

**DOI:** 10.1371/journal.pone.0196902

**Published:** 2018-05-15

**Authors:** Pankaj Kumar, Erika R. Belchamber, Stanley J. Miklavcic

**Affiliations:** Phenomics and Bioinformatics Research Center, School of Information Technology and Mathematical Sciences, University of South Australia, Mawson Lakes, Adelaide, Australia; Delft University of Technology, NETHERLANDS

## Abstract

Ellipse fitting is a highly researched and mature topic. Surprisingly, however, no existing method has thus far considered the data point eccentricity in its ellipse fitting procedure. Here, we introduce the concept of eccentricity of a data point, in analogy with the idea of ellipse eccentricity. We then show empirically that, irrespective of ellipse fitting method used, the root mean square error (RMSE) of a fit increases with the eccentricity of the data point set. The main contribution of the paper is based on the hypothesis that if the data point set were pre-processed to strategically add additional data points in regions of high eccentricity, then the quality of a fit could be improved. Conditional validity of this hypothesis is demonstrated mathematically using a model scenario. Based on this confirmation we propose an algorithm that pre-processes the data so that data points with high eccentricity are replicated. The improvement of ellipse fitting is then demonstrated empirically in real-world application of 3D reconstruction of a plant root system for phenotypic analysis. The degree of improvement for different underlying ellipse fitting methods as a function of data noise level is also analysed. We show that almost every method tested, irrespective of whether it minimizes algebraic error or geometric error, shows improvement in the fit following data augmentation using the proposed pre-processing algorithm.

## Introduction

The task of identifying and fitting ellipses to point data is an important and recurring problem in the mathematical and computer sciences, with a broad spectrum of applications. In his *Principia* (Book I, Section IV, Propositions 22-27) [1], Newton outlined how one could establish, precisely, the unique ellipse satisfying five pieces of information, either passing through given points or being tangent to given lines. The intention then was, as is historically documented, to determine the shape of orbits of planets and comets. Factored into the validity of those ellipses was, of course, the assumed accuracy of the observed input data. Naturally, uniqueness immediately becomes questionable when there are more than five pieces of independent data to fit, especially when each data point possesses some degree of measurement or observation error.

Applications of ellipse or conic fitting continue to arise to the present day not only in astronomy and astrophysics, e.g. in the study of galaxies [2], but also in camera optics, such as in the calibration of catadioptric cameras in [3] and [4], and with pin hole cameras, for the geometry of single axis rotatory motion [5] and [6]. In image analysis, ellipse fitting sees application in foreground segmentation, of biological cells in microscopic images [7] or of cereal grains in macroscopic images [8], and for the 3D reconstruction of root architecture [9, 10]. In the medical diagnosis of malaria, ellipse fitting was employed by Sheikhhosseini *et al.* [11], and more generally by Tang *et al.* in [12]. Ellipse fitting arises in biometrics, exemplified by the application to iris segmentation and localization [13–15] face detection [16] and pathological brain detection [17]. Finally, ellipse fitting arises in the application to industrial inspection [18] and control of silicon single crystal growth [19, 20].

Previous numerical approaches to ellipse fitting have usually focused on minimizing a distance function as a condition to satisfy in order to obtain the best fit to point data (see works by Rosin [21, 22]). In [23], Fitzgibbon *et al.* reported on a direct method based on minimizing an algebraic distance measure, while Halir and Flusser presented a numerically stable version of the same in [24]. A contrasting approach was followed by Ahn *et al.* [25, 26] who employed a geometric distance measure in their minimization scheme. A geometric distance measure featured also in a maximum likelihood estimation algorithm in [27, 28]. Other measures include treating ellipse data points as a noisy signal and applying filtering techniques [29], use of Gaussian Mixture Models [30], projective invariants [31] and hybrid approaches [18, 32]. In yet another approach, Kanatani and Rangarajan proposed hyperaccurate methods of ellipse fitting in [33] and [34], while Yu *et al.* in [35] proposed a new distance metric based on some intrinsic properties of ellipses and spheroids. Their new distance function had a clear geometric interpretation and was less computationally intensive than the geometric distance measure.

Despite these developments the majority of the ellipse fitting approaches had not considered a non-uniform weighting of individual contributions to the respective distance measures of error, thus taking particular account of the concept, which is introduced here, of *eccentricity of data points*. It is not difficult to imagine, however, that points which are more distant from the ellipse center and lying closer to the semi-major axis are more difficult to capture than data points which are closer to the ellipse center. In this paper we amend this deficiency by considering, in distance measures and ellipse fitting generally, a weighting of points according to their respective eccentricity values. We show that by doing so the performance of any fitting procedure is improved. Our mathematical definition of *point eccentricity* is given in the following Section. We note that Yu *et al.* [35] also considered including weightings in their optimization method, thus making their scheme more robust. Although somewhat related to their method, we argue that no new error measure is needed; existing measures, with their established advantages and disadvantages, are adequate but can be improved by a pre-processing of point data to achieve a better ellipse fit. In [21, 22] Rosin investigated different error functions which can be used in the least square fitting of ellipses. Among other factors Rosin assessed the suitability of various error functions against curvature bias. His objective was to gain better understanding of the merits of different EOF functions. Our objective, however, differs in that we focus on improving the performance of different ellipse fitting algorithms by an appropriate pre-processing of the raw data. Incidentally, in [22] Rosin concluded that most of the error functions he considered were insensitive to ellipse eccentricity. From this we again surmise that an approach such as the one we present here is more warranted rather than a consideration of alternative error measures. Our pre-processing of data is analogous to the resampling algorithm of particle filters where samples (data points) having a higher weighting are repeated and samples with insignificant weights are dropped. Through a series of numerical studies we show that residual errors of best ellipse fitting are reduced after processing and resampling of data points.

## Methods

In this section we introduce a mathematical definition of eccentricity of individual data points and demonstrate how the root mean square error (RMSE) of a fit increases with the average eccentricity of data points. The mean square error (MSE) calculation is based on an error measure that uses the shortest orthogonal distance of a point to the ellipse. The detailed description of this can be found in [36]. In the following subsection we present our modelling and simulation framework to validate our empirical observation and support the hypothesis that augmenting existing high eccentricity data points will improve the fit. In the final subsection we present our data supplementation algorithm, which is analogous to the resampling algorithm of particle filter [37].

### Eccentricity of a data point set

**Fig 1:**
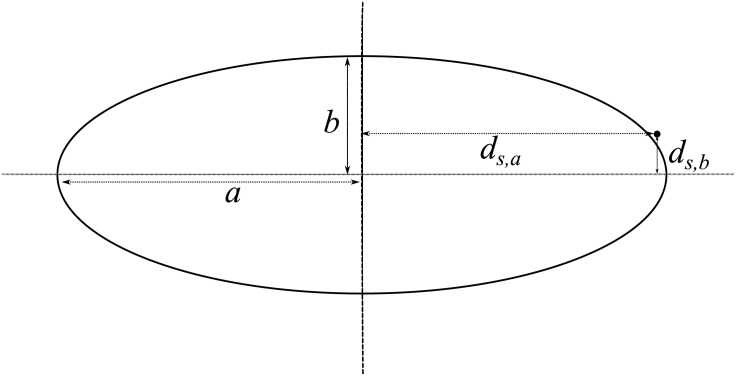
Schematic illustrating the eccentricity of an ellipse and of a data point as used in this paper.

The eccentricity of an ellipse is defined by $\varepsilon =\sqrt{\frac{a^{2}-b^{2}}{a^{2}}}$, where *a* is the semi-major axis and *b* is the semi-minor axis of the ellipse and 0 ≤ *ε* < 1. The value *ε* = 0 corresponds to a circle and the value *ε* = 1 corresponds to a straight line. Given a candidate ellipse with *a* and *b* and the orientation of corresponding axes defined, we here introduce the concept of pointwise eccentricity of a data set. For a point *X*_*s*_ of *S* points we define its eccentricity by the function,

$$\begin{array}{c} \xi_{s}=\varepsilon \left(\frac{d_{s,a}}{d_{s,b}+d_{s,a}}\right),\;s=1,\ldots ,S, \end{array}$$ (1)

where *d*_*s*,*a*_ is the orthogonal distance of data point *X*_*s*_ to the minor axis and *d*_*s*,*b*_ is the orthogonal distance of the point to the major axis (see Fig 1). This function, whose values range between 0 and 1, takes on larger values for data points that are more distant from the minor axis than from the major axis.

### Empirical evidence of increasing error with increasing eccentricity

**Fig 2:**
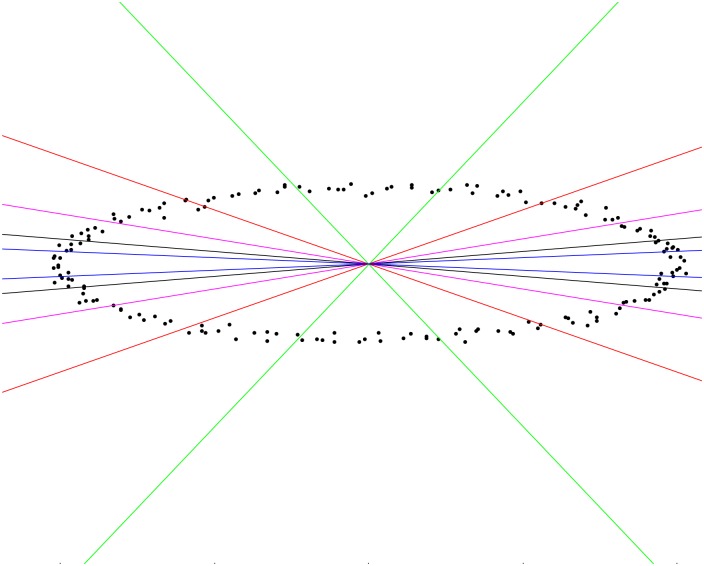
The angular regions between like-coloured lines (on the left and right of the ellipse centre) define sectors containing different data points for experimentation. The angular ranges are as follows: blue: [−4°, 4°] and [176°, 184°]; black:[−8°, 8°] and [172°, 188°]; magenta: [−15°, 15°] and [165°, 195°]; red: [−30°, 30°] and [150°, −210°]; green: [−60°, 60°] and [120°, 240°].

We conducted experiments to demonstrate that the RMSE of a fit increases with the average eccentricity of the data point set, $\bar{\xi }=\sum_{j=1}^{N}\xi_{j}/N$. In these experiments we generated a simulated set of data points to be fitted based on a random selection of points on a parametric ellipse and adding zero mean Gaussian noise to these. Different ellipse fitting algorithms were applied to obtain an ellipse of best fit to these data points. In our study we considered the following ellipse-fitting algorithms for which codes have been provided by the respective authors: CGIP-1979 [38], a basic and very initial approach to conic fitting; PAMI-1999 [23] and WSCG-1998 [24], are methods which minimize algebraic errors; PAMI-1991 [39], minimizes geometric distance error; and ECCV-2012 [28], is a MLE-based approach to minimize geometric distance. One could argue that a wider range of ellipse fitting algorithms should be considered for experimentation. However, those algorithms chosen represent a good cross-section of openly available ellipse fitting procedures. With our list we have covered both traditional as well as the latest methods; the set is representative of methods which minimize algebraic errors as well as those that minimize geometric errors. The computed RMSE values corresponding to data points within the various angular sectors of the conic as shown in Fig 2 are given in Table 1.

**Table 1:** Average RMSE value per data point in the different sectors for an ellipse of eccentricity 0.947418 with white noise of *σ* = 0.2. The RMSE increases with average eccentricity of the data point set. RMSE values are averages of 250 repeats for each method. Ranges of the data points are as follows: range 1: [−60°, 60°] and [120°, 240°]; range2: [−30°, 30°] and [150°, 210°]; range3: [−15°, 15°] and [165°, 195°]; range4:[−8°, 8°] and [172°, 188°]; range5: [−4°, 4°] and [176°, 184°].

Ellipse fitting methods	Average eccentricity of data points $\bar{\xi }$ in different sectors and corresponding angle ranges of the sectors				
	0.792539	0.872694	0.909291	0.926683	0.936820
	range1	range2	range3	range4	range5
CGIP-1979	3.774845	4.279448	4.739379	4.873605	4.906938
PAMI-1991	16.850676	19.962282	22.702475	23.489765	23.684542
PAMI-1999	18.154369	21.507089	24.459577	25.307857	25.517723
WSCG-1998	12.989561	15.384374	17.493729	18.099821	18.249772
ECCV-2012	14.750664	19.311192	19.933120	20.087211	20.133998

Data points in the different sectors result in different average eccentricities. The average eccentricity of data points decreases from the greatest value in the narrowest sector to the least value for points in the broader sector; in the former region, the data points are further from the ellipse centre.

**Fig 3:**
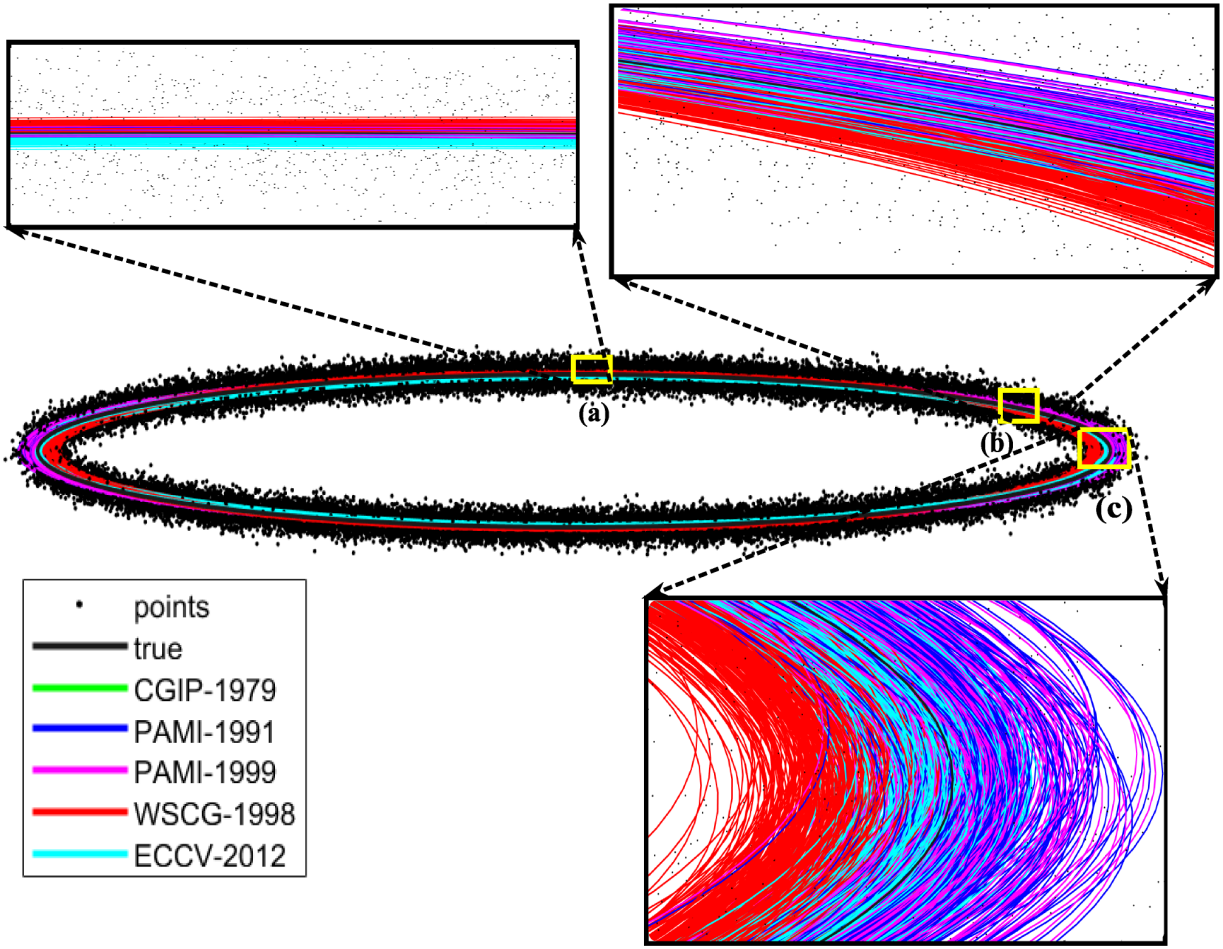
Synthesised data point set generated by introducing Gaussian noise to a given ellipse (gray solid curve). Other solid lines are fitting attempts using the methods listed in Table 1. Subplots are magnified views of regions (a), (b), and (c).

The literature clearly documents the fact that different ellipse fitting methods result in different average RMSE values. Naturally, in any application the foremost consideration is the desire to employ a method that gives a lowest RMSE. However, factors other than the lowest RMSE value may come into consideration; there may be other application-specific criteria, such as computational efficiency, that influence the choice of one method over another. All the same, in our experiments, computing RMSE values for data points with different average eccentricities, we clearly see a trend of increasing RMSE with increasing average eccentricity of the data point set, see Table 1; this trend, moreover, is common to all ellipse fitting methods. This phenomena is visually demonstrated in Fig 3, which shows magnified views of the results of ellipse fitting in regions of different mean eccentricity: errors are higher for the more eccentric data points. The results given in Table 1 and shown in Fig 3, are averages over 250 repeats for each of the five different algorithms.

### Ellipse data augmentation algorithm

Resampling is a process used in particle filters to avoid the problem of particle degeneration [37]. In that application particles having greater weights are repeated while particles with insignificant weights are dropped, with the overall number of particles being preserved. Algorithm 1 gives the pseudo code for the present augmentation method where, in contrast to the usual resampling process, we increase the data point set by increasing the number of points in the vicinity of those points having high point-wise eccentricities. Denote by *X*_*s*_ = (*x*_*s*_, *y*_*s*_) an arbitrary (2D) data point in a set of *S* points, *s* = 1, …, *S*. To each such data point we assign a weight according to the function

$$\begin{array}{c} w_{s}=e^{\xi_{s}}, \end{array}$$ (2)

where *ξ*_*s*_ is the point’s eccentricity as defined in Eq (1). The augmented sampling algorithm described in Algorithm 1 is applied to the data point set complemented by the set of corresponding normalized weights

$$\begin{array}{c} W_{s}=w_{s}/\sum_{k=1}^{S}w_{k}. \end{array}$$ (3)

**Algorithm 1**: Given discrete set ${\left\{\left(X_{s},W_{s}\right)\right\}}_{s=1}^{S}$, of *S* data points *X*_*s*_ with weights *W*_*s*_, by adding supplementary points produce a discrete set ${\left\{\left(Y_{t},Z_{t}\right)\right\}}_{t=1}^{T}$ of *T* data points *Y*_*t*_ with weights *Z*_*t*_ such that *T* ≥ *S*.

1. Perform an ellipse fit to the *S* data points ${\left\{X_{s}\right\}}_{s=1}^{S}$; *a* and *b* are defined.

2. Assign an eccentricity value, *ξ*_*s*_, to each data point according to Eq (1).

3. Compute weights ${\left\{W_{s}\right\}}_{s=1}^{S}$ for data point set ${\left\{X_{s}\right\}}_{s=1}^{S}$ according to Eqs (2) and (3).

4. Set *T* = *integer*((*min*(*W*_*s*_))^−1^) > *S*.

5. Construct the set of cumulative weights, C: *c*_1_ = *W*_1_,

**for** *s* = 2: *S* **do**

 construct C: *c*_*s*_ = *c*_*s*−1_ + *W*_*s*_

**end for**

6. Initialize variables *t*_*old*_ = 1, *increment*_*s*_ = *false*, *X*_*s*_*old*__ = *X*_1_ and *μ*_1_ = 0 or *T*^−1^.

7. Begin: *s* = 1 (the base of C)

**for** *t* = 1: *T* **do**

 Move along C: *μ*_*t*_ = *μ*_1_ + *T*^−1^(*t* − 1)

 **while** (*μ*_*t*_ > *c*_*s*_) **do**

  *X*_*s*_*old*__ = *X*_*s*_;

  *s* = *s* + 1;

  *increment*_*s*_ = *true*;

 **end while**

 **if** *increment*_*s*_ == *true* **then**

  {*Y*_*t*_,…, *Y*_*t*_*old*__} = interpolate ([*X*_*s*_, *X*_*s*_*old*__] for {*t*_*old*_, …, *t*});

  Assign new weights *Z*_*u*_ = *T*^−1^, *u* ∈ {*t*_*old*_, …, *t*};

  *t*_*old*_ = *t*;

  *increment*_*s*_ = *false*.

 **end if**

**end for**

In our application of the augmented sampling algorithm, the numbers of input data points and output data points can be varied. In practice, better ellipse fitting results when the number of output data points exceeds the number of input data points. As already mentioned, we have adopted the strategy of retaining all original data points and augmenting the set with new points in the region around those data points having higher weights. To increase the data point set the additional data points are obtained (in the sequential Algorithm (1)) by interpolating between point currently considered and its preceding neighbour. The number of points to be added by interpolation is set by the number of 1/*T* steps required to cross the current value *c*_*s*_ in the cumulative weight distribution. To compute the weights of data points a knowledge of the major and minor axis is required. In our experiments using the synthesized data set referred to in the subsection *Eccentricity of a data point set*, the major and minor axes are known from the original ellipse used to generate the data points. In the example application involving a series of real data sets, an example of which is depicted in subsection *Application to a root phenotyping data set*, of the *Results and Discussion* section, an estimate of the major and minor axes was obtained by taking means of ellipse parameters generated using two or more of the five different methods. Alternatively, one could take two passes of the same algorithm before and after data augmentation. The estimation of the eccentric weights of the data points is not sensitive to small errors in the estimate of the major and minor axes.

## Theoretical model and simulations

In the preceding section we provided an empirical demonstration of the effect of data point eccentricity on the accuracy of a fit to observed data. We also described an algorithm used to generate a supplementary set of data points that we hypothesize would improve the accuracy. Before presenting numerical results of simulations in the next section we shall here provide a theoretical validation of the hypothesis as well as indicate the quantitative limitations to an augmentation process. In other words, we establish here, albeit for an ideal setting, criteria that need to be satisfied for improved fitting.

There are different parametric representations of an ellipse. An ellipse lying in the *x*′ *y*′–plane can be represented by the (generic) conic equation

$$\begin{array}{c} Ax^{\prime 2}+Bx^{\prime }y^{\prime }+Cy^{\prime 2}+Dx^{\prime }+Ey^{\prime }+F=0, \end{array}$$ (4)

with the constraint *B*^2^ − 4*AC* = 1. Eccentricity of the ellipse, based on the coefficients (*A*, *B*, *C*, *D*, *E*, *F*), is then given by

$$\begin{array}{c} \varepsilon =\frac{2\sqrt{{\left(A-C\right)}^{2}+B^{2}}}{\eta \left(A+C\right)+\sqrt{{\left(A-C\right)}^{2}+B^{2}}}, \end{array}$$ (5)

where *η* = 1 when $|\begin{array}{ccc} A & B/2 & D/2\\ B/2 & C & E/2\\ D/2 & E/2 & F \end{array}|<0$ and *η* = −1 otherwise. Alternatively, an ellipse with semi-major and semi-minor axes (*a*_0_, *b*_0_), centered at (*x*_0_, *y*_0_) and with major axis oriented an angle *θ* relative to the *x*′ axis can be represented by

$$\begin{array}{c} \left(\frac{{\left(\left(x^{\prime }-x_{0}\right)cos\theta +\left(y^{\prime }-y_{0}\right)sin\theta \right)}^{2}}{a_{0}^{2}}\right)+\left(\frac{{\left(\left(y^{\prime }-y_{0}\right)cos\theta +\left(x^{\prime }-x_{0}\right)sin\theta \right)}^{2}}{b_{0}^{2}}\right)=1 \end{array}$$ (6)

### Construction of ground truth and observation data

Through suitable affine transformations, (*x*′, *y*′)→(*x*, *y*), the ellipse center at (*x*_0_, *y*_0_) can be mapped to the origin (0, 0) and *θ* is mapped to 0. Thus, without loss of generality but with the advantage of simplicity, an ellipse, *E*_0_, can be represented as

$$\begin{array}{c} E_{0}=\left\{\left(x,y\right):x^{2}/a_{0}^{2}+y^{2}/b_{0}^{2}=1\right\}. \end{array}$$ (7)

With this description, *E*_0_ will be here used to define ground truth data, which we shall attempt to approximate after some treatment with noise and data point addition.

We choose *N* points, ${\left\{\left({\bar{x}}_{i},{\bar{y}}_{i}\right)\right\}}_{i=1}^{N}$, from that part of *E*_0_ which lies in the first quadrant. The points ${\left\{\left({\bar{x}}_{i},{\bar{y}}_{i}\right)\right\}}_{i=1}^{N}$ can be presented in polar co-ordinates as ${\bar{x}}_{i}=r_{i}cos\theta_{i}$ and ${\bar{y}}_{i}=r_{i}sin\theta_{i}$, where *r*_*i*_, *θ*_*i*_ are the polar co-ordinates of the point $\left\{\left({\bar{x}}_{i},{\bar{y}}_{i}\right)\right\}$. These points are then given random *small* perturbations (not necessarily zero mean) to obtain a set *C*′ of new points ${\left\{\left({\tilde{x}}_{i},{\tilde{y}}_{i}\right)\right\}}_{i=1}^{N}$. These perturbed data points lie randomly about *E*_0_. Using this set we generate a set of 4*N* points by first reflecting the set *C*′ about the *x*–axis to give a second set *C*′′, and then reflecting both *C*′ and *C*′′ about the *y*–axis to give new sets *C*′′′ and *C*′′′′, respectively. This simple reflective operation ensures, that the centre of the fitted ellipse will conveniently be the origin of the *xy*–coordinate system. Thus, any contribution to the error of a fit will only be due to errors in the estimates of the semi-major and semi-minor axes of the fitted ellipse.

We now seek to obtain an ellipse of best fit, $E_{1}=\left\{\left(x,y\right):x^{2}/a_{1}^{2}+y^{2}/b_{1}^{2}=1\right\}$, to the combined random data set *C* = *C*′∪*C*′′∪*C*′′′∪*C*′′′′, where the positive constants *a*_1_ and *b*_1_ are chosen to minimize the mean square error,

$$\begin{array}{ccc} \Sigma & = & \frac{1}{N}\sum_{i=1}^{N}\left[{\left(x\left(\theta_{i}\right)-x_{i}\right)}^{2}+{\left(y\left(\theta_{i}\right)-y_{i}\right)}^{2}\right],\\ & = & \frac{1}{N}\sum_{i=1}^{N}\left[{\left(a-r_{i}\right)}^{2}\cos^{2}\theta_{i}+{\left(b-r_{i}\right)}^{2}\sin^{2}\theta_{i}\right], \end{array}$$ (8)

In the above, because of the doubly reflective symmetry of the data points, we need only actively consider the *N* original data points of *C* lying in the first quadrant. The commonality of *θ* values in the polar representation of the ground truth data points allows easy identification of corresponding points on the parametric fitted ellipse and thus ready formulation of a simple algebraic representation of the geometric error Eq (8).

We minimize (8) in the usual way and solve the equations generated by the zero derivatives, ∂Σ/∂*a* = 0 and ∂Σ/∂*b* = 0, to obtain the best constants *a*_1_ and *b*_1_:

$$\begin{array}{c} a_{1}=\frac{\sum_{i=1}^{N}r_{i}\cos^{2}\theta_{i}}{\sum_{i=1}^{N}\cos^{2}\theta_{i}}\;\text{and}\;b_{1}=\frac{\sum_{i=1}^{N}r_{i}\sin^{2}\theta_{i}}{\sum_{i=1}^{N}\sin^{2}\theta_{i}}. \end{array}$$ (9)

A measure of the error arising from this approximation, *E*_1_, to the true ellipse, *E*_0_, is given by the *L*_2_ norm

$$\begin{array}{ccc} \Sigma_{1} & = & \int_{0}^{2\pi }\left[{\left(a_{0}-a_{1}\right)}^{2}\cos^{2}\theta +{\left(b_{0}-b_{1}\right)}^{2}\sin^{2}\theta \right]d\theta \\ & = & \pi [{\left(a_{0}-a_{1}\right)}^{2}+{\left(b_{0}-b_{1}\right)}^{2}]. \end{array}$$ (10)

Our aim now, according to our hypothesis, is to improve on this error by adding supplementary data points to the original data set. That this is indeed possible is readily shown by adding particular points to our original set, *C*, noting that, by construction, we do so in such a way as to maintain symmetry. Two possibilities arise. First, one may introduce 2*n* new points on the *x*–axis, *n* at *θ* = 0 and *n* corresponding points at *θ* = *π*. Alternatively, we introduce *m* new off-axis points in the positive quadrant and generate symmetric reflections of these points in the other three quadrants, giving a total of 4*m* new sample points. However, it suffices for our analysis to consider the simple cases of adding a single point in each of the above two scenarios. That is, *n* = 1 and *m* = 1, respectively.

### Supplementary point on-axis

We consider first the case of a single new point at *θ*_*N*+1_ = 0 (as well as its mirror image point at *θ*_*N*+2_ = *π*). From symmetry, in the coming analysis we need only consider the point at *θ*_*N*+1_ = 0. The least squares procedure results in a new best-fit ellipse, *E*_2_, with optimal ellipse parameters given by

$$\begin{array}{c} a_{2}=\frac{\sum_{i=1}^{N+1}r_{i}\cos^{2}\theta_{i}}{\sum_{i=1}^{N+1}\cos^{2}\theta_{i}}\;\text{and}\;b_{2}=\frac{\sum_{i=1}^{N+1}r_{i}\sin^{2}\theta_{i}}{\sum_{i=1}^{N+1}\sin^{2}\theta_{i}}. \end{array}$$ (11)

That is,

$$\begin{array}{c} a_{2}=\frac{\sum_{i=1}^{N}r_{i}\cos^{2}\theta_{i}+R}{\sum_{i=1}^{N}\cos^{2}\theta_{i}+1}\;\text{and}\;b_{2}=\frac{\sum_{i=1}^{N}r_{i}\sin^{2}\theta_{i}}{\sum_{i=1}^{N}\sin^{2}\theta_{i}}\equiv b_{1}, \end{array}$$ (12)

where we have written *R* for *r*_*N*+1_. The error incurred by this new approximation is thus

$$\begin{array}{c} \Sigma_{2}=\pi \left[{\left(a_{0}-a_{2}\right)}^{2}+{\left(b_{0}-b_{2}\right)}^{2}\right]. \end{array}$$ (13)

The difference between the errors in these two approximations,

$$\begin{array}{c} \Delta \Sigma \equiv \Sigma_{1}-\Sigma_{2}=\pi \left[{\left(a_{0}-a_{1}\right)}^{2}-{\left(a_{0}-a_{2}\right)}^{2}\right], \end{array}$$ (14)

is our point of focus. If we denote by *β* the ratio

$$\begin{array}{c} \beta =\frac{\sum_{i=1}^{N}\cos^{2}\theta_{i}+R/a_{1}}{\sum_{i=1}^{N}\cos^{2}\theta_{i}+1}, \end{array}$$ (15)

then this difference in error becomes

$$\begin{array}{c} \Delta \Sigma =\pi \left(\beta -1\right)a_{1}\left[2a_{0}-\left(\beta +1\right)a_{1}\right]. \end{array}$$ (16)

Consequently, the new least squares fit obtained by adding a supplementary point on the (semi-major) axis will be an improvement provided ΔΣ > 0 or, equivalently, if the two factors appearing in Eq (16) are of the same sign. This condition can be shown to reduce to the summary inequality condition on the distance from the origin of the supplementary point in terms of given information,

$$\begin{array}{c} \min\left\{a_{1},a_{1}+\Delta a_{0}\right\}<R<\max\left\{a_{1},a_{1}+\Delta a_{0}\right\}. \end{array}$$ (17)

In Eq (17) we have introduced

$$\begin{array}{c} \Delta a_{0}=2\left(a_{0}-a_{1}\right)\left[\sum_{i=1}^{N}\cos^{2}\theta_{i}+1\right], \end{array}$$ (18)

in terms of given information. The case of *a*_1_ being the minimum of the two scalar values corresponds to the case *a*_1_ < *a*_0_, suggesting that the randomized data points lie predominantly within the original ellipse, *E*_0_, while the case of *a*_1_ being the maximum is correlated with *a*_1_ > *a*_0_ and the randomized points lying beyond the original ellipse.

The first and obvious conclusion to draw from this result is that there exists a two-sided constraint on where an additional point can be placed for there to be improvement (relative to the ground truth). Adding a point (or points) too close to the origin or two far away will result in a less accurate fit. The second conclusion is that the restricted placement is dependent not only on the observed data to be fitted, but also on that data’s relation to the true ellipse position. Indeed, the limited choice for *R* nevertheless seeks to counteract the inclination of the noisy data to either under- or overestimate the ellipse’s size and eccentricity. A third conclusion is that Eq (17) verifies our hypothesis that data augmentation can improve the fitted result.

### Supplementary point off-axis

We shall now consider the more general and more likely case of additional off-axis points. In particular, we consider adding the supplementary point *X*_*N*+1_ = (*x*_*N*+1_, *y*_*N*+1_) = (*R* cos *α*, *R* sin *α*). Although we make no assumptions about the values of *α*, it is worth noting that, according to our intent, *X*_*N*+1_ will be a high eccentricity point. Thus, *α* will be close to zero. The new least squares fit will possess the optimal ellipse parameter values

$$\begin{array}{ccc} a_{2} & = & \frac{\sum_{i=1}^{N}r_{i}\cos^{2}\theta_{i}+R\cos^{2}\alpha }{\sum_{i=1}^{N}\cos^{2}\theta_{i}+\cos^{2}\alpha }=a_{1}\beta , \end{array}$$ (19)

$$\begin{array}{ccc} b_{2} & = & \frac{\sum_{i=1}^{N}r_{i}\sin^{2}\theta_{i}+R\sin^{2}\alpha }{\sum_{i=1}^{N}\sin^{2}\theta_{i}+\sin^{2}\alpha }=b_{1}\delta , \end{array}$$ (20)

where

$$\begin{array}{c} \beta =\frac{\sum_{i=1}^{N}\cos^{2}\theta_{i}+R/a_{1}\cos^{2}\alpha }{\sum_{i=1}^{N}\cos^{2}\theta_{i}+\cos^{2}\alpha }\;\text{and}\;\delta =\frac{\sum_{i=1}^{N}\sin^{2}\theta_{i}+R/b_{1}\sin^{2}\alpha }{\sum_{i=1}^{N}\sin^{2}\theta_{i}+\sin^{2}\alpha } \end{array}$$ (21)

are defined in analogy to (and are generalizations of) the *β* in Eq (16). The difference in errors in the ellipse fittings is now expressed as

$$\begin{array}{c} \Delta \Sigma =\pi \{\left[{\left(a_{0}-a_{1}\right)}^{2}-{\left(a_{0}-a_{2}\right)}^{2}\right]+\left[{\left(b_{0}-b_{1}\right)}^{2}-{\left(b_{0}-b_{2}\right)}^{2}\right]\} \end{array}$$ (22)

or, more simply, as

$$\begin{array}{c} \Delta \Sigma =\pi \{\left(\beta -1\right)a_{1}\left[2a_{0}-\left(\beta +1\right)a_{1}\right]+\left(\delta -1\right)b_{1}\left[2b_{0}-\left(\delta +1\right)b_{1}\right]\}. \end{array}$$ (23)

For the fitting to be an improvement we again require a positive difference, *i.e.*, ΔΣ > 0. Not surprisingly, this condition leads to a greater number of possible cases than was identified in the previous subsection. In fact, there are twelve possible combinations of the four factors appearing in Eq (23) that result in a positive ΔΣ (*e.g.*, both terms being positive can either mean that all factors are positive, the first two are positive while the second two are negative, the reverse case, or all four factors being negative).

A systematic study will show that these twelve possible combinations correspond to four scenarios associated with different distributions of the randomized data points relative to the true ellipse. Framing the discussion in terms of the points in the first quadrant only (the remainder follow from symmetry), the scenarios are as follows.

Type 1. the set of random points (0 < *θ*_*i*_ < *π*/2, *i* = 1, …, *N*) lie predominantly within the original ellipse,
Type 2. the points lie predominantly outside the original ellipse,
Type 3. points near *θ* = *π*/2 lie within the original ellipse, while those near *θ* = 0 lie predominantly beyond the original ellipse, and
Type 4. points near *θ* = *π*/2 lie outside the original ellipse, while those near *θ* = 0 lie predominantly within the original ellipse.

In fact, it can be shown that of the twelve possible combinations of terms and factors, leading to a non-negative ΔΣ, four fall uniquely into one scenario, while the other eight alternative combinations correspond to two scenarios. Consequently, associated with each of the four scenarios are five combinations of the terms and factors in Eq (23). Of the twelve cases, six can be eliminated immediately as they violate one or more fundamental conditions (*e.g.*, *b*_1_ > *R* simultaneously as *R* > *a*_1_, which contradicts our ellipse construction with *b*_1_ < *a*_1_). After some elementary algebra, the remaining six cases can be reduced to the following summary conditions

$$\begin{array}{c} b_{1}<R<b_{1}+\Delta b_{\alpha }\;\;\;\text{and}\;\;\{\begin{array}{cc} & R<\min\{a_{1},a_{1}+\Delta a_{\alpha }\}\\ & \min\left\{a_{1},a_{1}+\Delta a_{\alpha }\right\}<R<\max\left\{a_{1},a_{1}+\Delta a_{\alpha }\right\}\\ & R>\max\{a_{1},a_{1}+\Delta a_{\alpha }\} \end{array} \end{array}$$ (24)

for the case of Δ*b*_*α*_ > 0, or

$$\begin{array}{c} \min\left\{a_{1},a_{1}+\Delta a_{\alpha }\right\}<R<\max\left\{a_{1},a_{1}+\Delta a_{\alpha }\right\}\;\text{and}\;R>\max\left\{b_{1},b_{1}+\Delta b_{\alpha }\right\} \end{array}$$ (25)

for Δ*a*_*α*_ and Δ*b*_*α*_ positive or negative. Here we have introduced

$$\begin{array}{c} \Delta a_{\alpha }=\frac{2\left(a_{0}-a_{1}\right)\left[\sum_{i=1}^{N}\cos^{2}\theta_{i}+\cos^{2}\alpha \right]}{\cos^{2}\alpha } \end{array}$$ (26)

and

$$\begin{array}{c} \Delta b_{\alpha }=\frac{2\left(b_{0}-b_{1}\right)\left[\sum_{i=1}^{N}\sin^{2}\theta_{i}+\sin^{2}\alpha \right]}{\sin^{2}\alpha }. \end{array}$$ (27)

Note that Δ*b*_*α*_ (Δ*a*_*α*_) can be large for *α* near 0 (*π*/2).

Inequality conditions, Eqs (24) and (25), are generalizations of Eq (17) to the case of an additional off-axis point. Similarly, Δ*a*_*α*_ and Δ*b*_*α*_ are generalisations of Δ*a*_0_.

Regardless of alternative, a pair of inequalities is to be satisfied simultaneously, which thus establishes allowed values of both *α* and *R* (not just *R*). The greater number of possibilities allowed through Eqs (24) and (25) is due to the added degree of freedom introduced with a nonzero *α*. Thus, as in the preceding case of an on-axis supplementary point, the above inequalities indicate that point additions cannot be made arbitrarily, but provided the points satisfy intuitive conditions they will lead to an improved fit.

**Fig 4:**
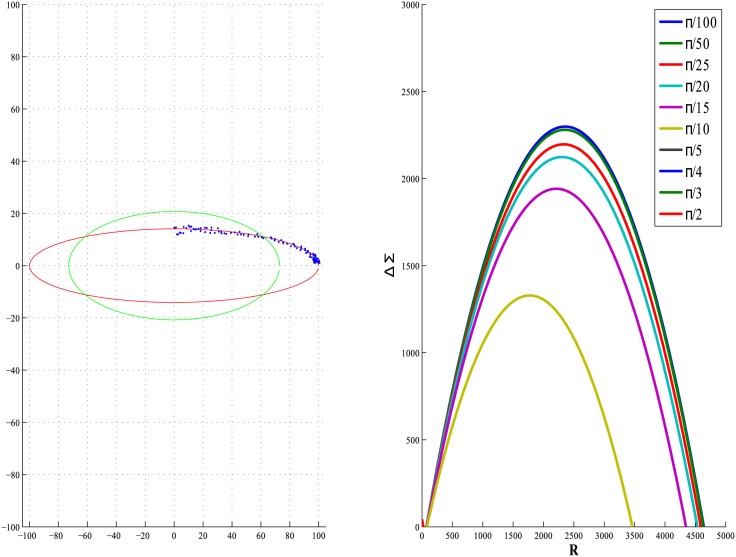
Plots of error ΔΣ verses R as a function of angle *α* for the Type 3 case, for an ellipse of eccentricity 0.99.

In Fig 4 we show results of ΔΣ as a function of continuously varying *R* for different values of *α* for the Type 3 scenario. The plots clearly show ΔΣ to be positive for a large range of *R* values especially for small values of *α*. Hence, strategically introducing replicate points in the regions occupied by high eccentricity data points will improve the fit of an ellipse. This fact is reinforced by the empirical exercise in the following section.

## Results and discussion

In this section we demonstrate the improvement achieved by the application of our data augmentation algorithm. We do so first by a quantitative evaluation of the error incurred in fitting. A second demonstration is by means of a visual improvement in an application of 3D reconstruction of root architecture for plant root phenotyping wherein ellipse fitting is a critical intermediate step to decipher camera parameters.

### Quantitative analysis of improved ellipse fitting

To evaluate the efficacy of our augmentation method we repeated the experiment described in Subsection *Empirical evidence of increasing error with increasing eccentricity*, with a second pass of the five different ellipse fitting methods. The RMSE values of the new fits are listed in Table 2 for an ellipse of eccentricity 0.9474. Zero-mean, Gaussian noise with a normalized standard deviation of 0.2 has again been added to each data point. The RMSE values before and after application of data augmentation for the case of PAMI-1999 [23] were 18.1445 and 11.9947, respectively, the method thus showing the largest overall improvement of 6.1597 (first column in Table 2) over the other methods, followed closely by PAMI-1991 [39], which showed a slightly lower improvement of 6.1205. In contrast, the RMSE improvement for the CGIP-1979 [38] method was not (as) significant, being only 0.0508, with the pre- and post-augmentation RMSE values being only 3.7748 and 3.7240, respectively. It is clear that little improvement can be expected in this case since this fitting procedure, even in its fundamental form, is able to capture the more eccentric points better than the other methods. Nevertheless, we can legitimately conclude that all five ellipse fitting methods showed improvement following pre-processing using the proposed algorithm.

**Table 2:** Average RMSE per data point in the different sectors for an ellipse of eccentricity 0.947418 with white noise of *σ* = 0.2. After pre-processing the data points with the data interjection algorithm proposed here. The RMSE over each sector has gone down compared to the results of RMSE per data point in Table 1 for the same sectors.

Ellipse fitting methods	Average eccentricity of data points $\bar{\xi }$ in different sectors and corresponding angle ranges of the sectors				
	0.792539	0.872694	0.909291	0.926683	0.936820
	range1	range2	range3	range4	range5
CGIP-1979	3.723994	4.115117	4.479227	4.586599	4.613332
PAMI-1991	10.730208	12.684557	14.408411	14.904043	15.026683
PAMI-1999	11.994722	14.178519	16.104849	16.658706	16.795754
WSCG-1998	8.846329	10.450755	11.866656	12.273833	12.374592
ECCV-2012	10.249949	13.144765	13.543470	13.642364	13.672400,

In plots of Figs 5 and 6, we present a more extensive comparison between fits prior to and following data augmentation as a function of data point noise level. Five different levels of noise were considered: *σ* = 0.1, 0.2, 0.3, 0.4, and 0.5. The curves in Figs 5 and 6 give the RMSE values (and their variances) following 250 repeats of random addition of noise to the data points followed by the fitting exercise. The RMSE values shown have been normalized, respectively, by the maximum RMSE values obtained by each fitting method. This shifts the focus to the relative improvement in the fit brought about by the proposed data augmentation algorithm, and suppresses the differences in absolute performance of the different algorithms. The RMSE values of the fits before application of the proposed algorithm are shown by dashed lines, while the outcomes following pre-processing are represented by solid lines. We note first that adding increasing levels of zero-mean, Gaussian noise to the data generally increases the RMSE, on average. However, in all cases of low magnitude noise (small *σ*), the pre-processing algorithm helps improve the quality of the fit as shown by the reduced RMSE values (Figs 5 and 6). The improvement is consistent at all levels of noise, *σ*, except for the high noise case of CGIP-1979 [38]. This may be due to the high noise sensitivity of this algorithm. However, as mentioned, at very high noise levels, the behavior of the fitting algorithms generally becomes of poorer quality.

**Fig 5:**
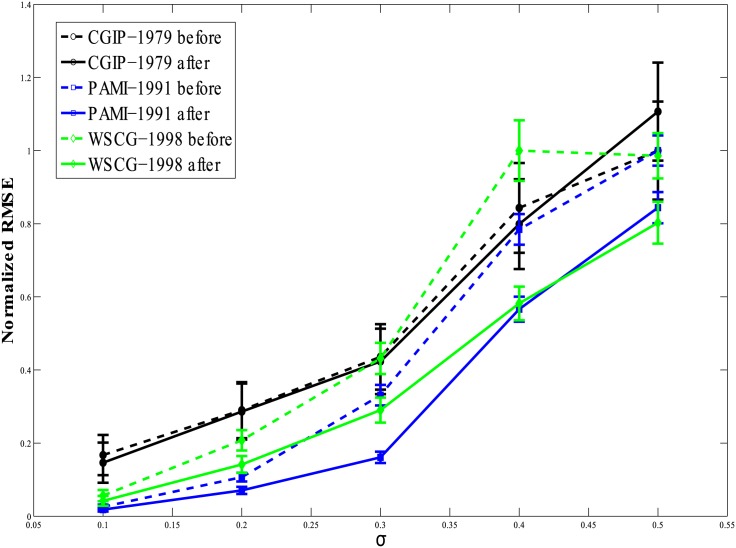
RMSE vs increasing noise level *σ* plot for CGIP-1979 in black, PAMI-1991 in blue and WSCG-1998 in green. The dashed line shows RMSE values prior to application of our pre-processing method while the solid line shows the RMSE values after application. The RMSE after application of the pre-processor to the data is lower than before in almost all cases.

**Fig 6:**
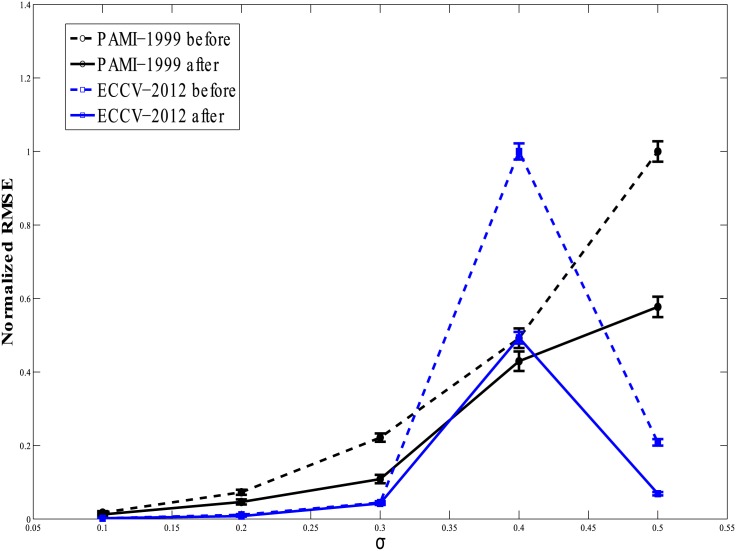
RMSE vs increasing noise level *σ* plot for PAMI-1999 in black and ECCV-2012 in blue. The dashed line shows RMSE values prior to application of our pre-processing method while the solid line shows the RMSE values after application. The RMSE after application of the pre-processor to the data is lower than before in all cases.

As with the comparison between the results in Tables 1 and 2, from these plots we can unequivocally conclude that the data pre-processing algorithm improves the performance of all fitting methods.

### Application to a root phenotyping data set

We applied the ellipse data pre-processing algorithm to a root phenotyping exercise. Corn plants were grown in a transparent gellan gum medium and imaged on a turntable platform. For analysis, 72 images of the root architecture were taken across a 360° rotation range. Viewed along the rotation axis, the rotation of a given root tip traces out a circle. However, in the image plane perspective of the camera system the trajectory appears as a highly eccentric ellipse. Ellipse fitting was applied to a series of such root tip trajectories in order to self-calibrate the camera system; the best camera calibration is achieved with the most accurate ellipse fits. Subsequently, a 3D reconstruction of the root system was performed using the foreground information derived from the image sequence. Of particular significance in the present context is the fact that a more accurate 3D reconstruction of the root architecture for quantitative phenotyping purposes is achieved with better camera calibration (and thus of better ellipse fitting). Between 9 and 15 ellipse fits were carried out for a given turntable image sequence.

**Fig 7:**
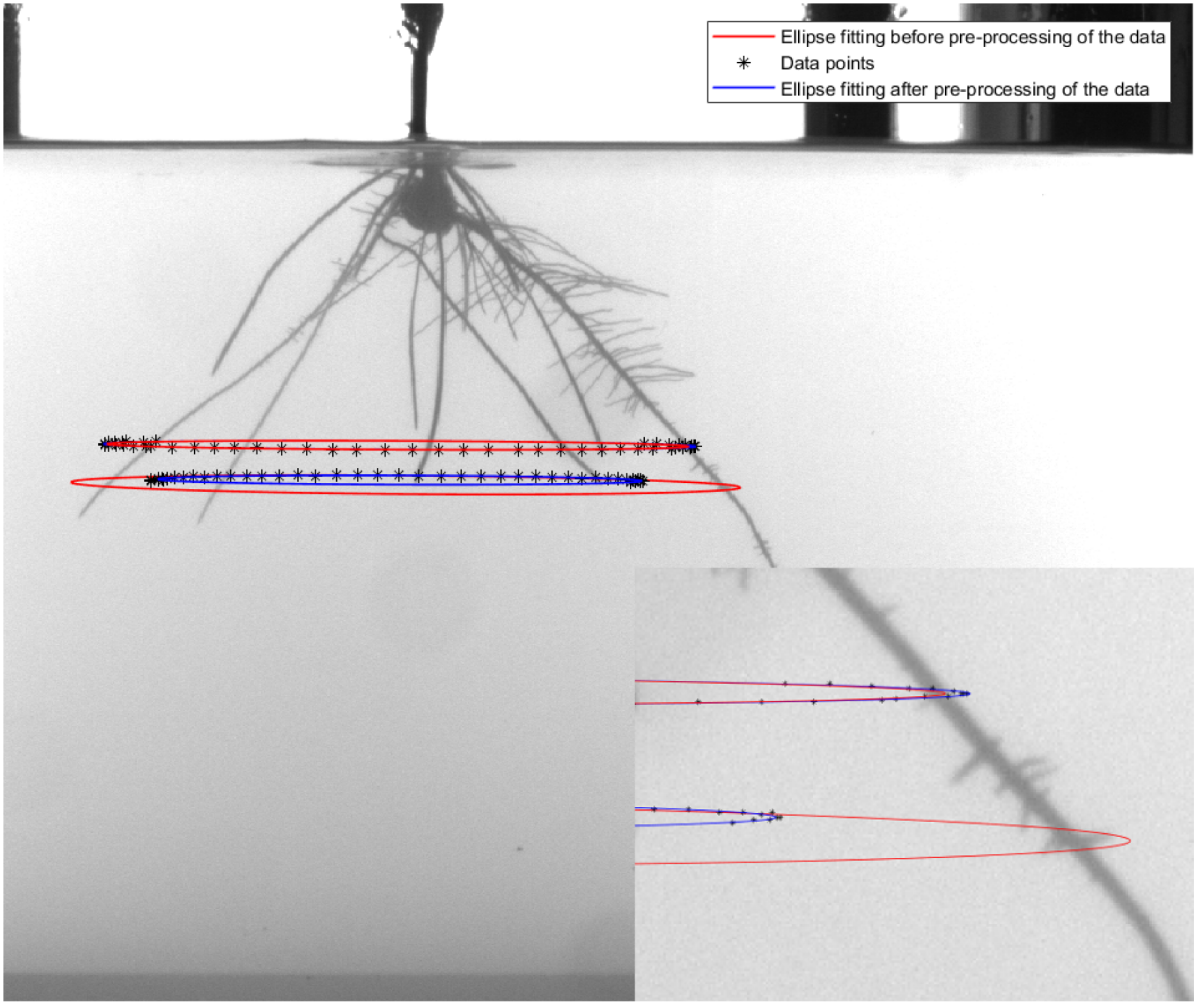
Sample image of a corn plant grown in a transparent medium of gellan gum at day 14. The black symbols depict incomplete data sets of captured locations of two root tips (identified as the tips in contact with the blue ellipses in the image centre) during a 360° rotation of turntable and 72 image acquisition procedure. The superimposed ellipses are results of ellipse fitting of the discrete data sets using the PAMI-1999 algorithm before (red curve) and after (blue curve) pre-processing of the data using the data augmentation algorithm proposed here.

Fig 7 shows an image of the plant root system at 14 day. Overlaid, firstly, are two discrete point sets (black symbols) of locations of two root tips (identified as the roots with tips touching the blue ellipses) that have been tracked through a 360° rotation of a turntable and imaged 72 times. Note that in each case some of the 72 data points are absent. Although tangential to the point of discussion, this incomplete data sets result (in this application) in each case from a single instance of occlusion of that particular tip by another root during the tracking of the former tip through the sequence of 72 images. Once the tracking process is unable to locate that particular tip in an image, all tip location data in subsequent images is lost. Although this technical problem can be resolved, the current method fortuitously provided us with incomplete sets for analysis. Also superimposed on the image are red ellipses which have been obtained using the PAMI-1999 algorithm applied to the raw data sets. It is clear that the method produces an exaggerated ellipse extending well beyond the range of the data in the lower set (disregarding the high curvature turnaround at the ends of the data set), and an ellipse that underestimates the data set in the upper set. In contrast, the ellipses in blue, obtained by fitting with the same method PAMI-1999 after pre-processing of the data with the proposed data augmentation algorithm faithfully follow the respective point sets. Visually it is clear that ellipse fitting to pre-processed data produces better results. This leads to a better 3D reconstruction of the root system architecture.

We remark that, depending on distance to the axis of rotation, occlusion can occur anywhere along the trajectory (including the high curvature regions at the ends). However, as evidenced by the present example, the data augmentation procedure remains effective provided there exist a sufficient number of data points in the vicinity of at least one of the two extremes of the major axis of the ellipse (where symmetry can be utilized to compensate for missing data at the other extreme). We may comment that the incomplete data sets shown in Fig 7 are comparable to the partial data considered by Fitzgibbon *et al.* [23], although the eccentricities of the implied ellipses here are considerably higher than supposed in their study.

**Fig 8:**
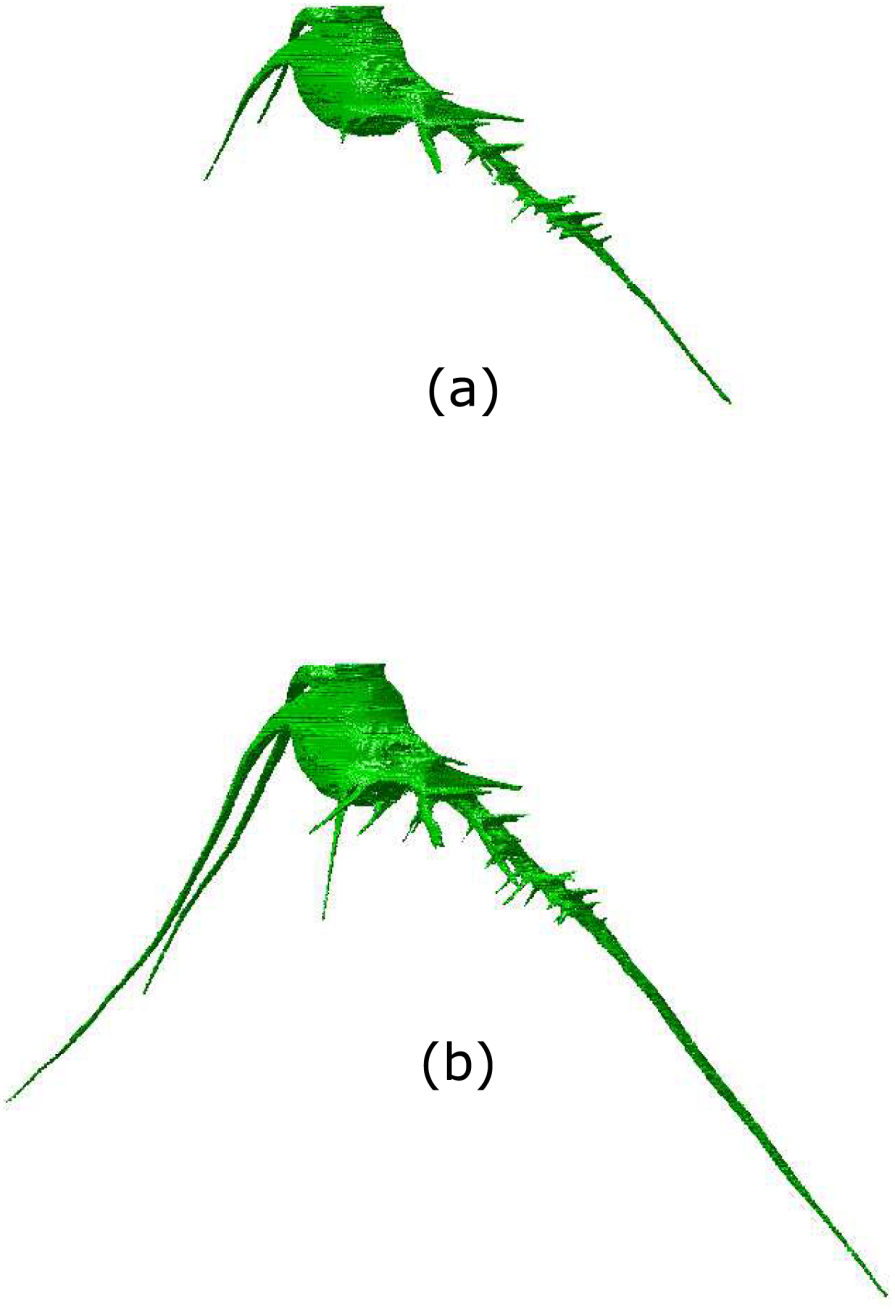
Image (a) shows a 3D reconstruction of a root system (using a visual hull algorithm) where the ellipse fitting step has been carried out without application of the data augmentation algorithm. Image (b), on the other hand, shows an improved 3D reconstruction as a consequence of the data augmentation algorithm in the ellipse fitting step.

Fig 8(a) shows the result of a 3D reconstruction of the root system without application of the ellipse data pre-processing algorithm, while Fig 8(b) shows the greatly improved 3D reconstruction following the application of our data augmentation method. Fig 8(b) clearly shows a more complete root system, which would allow the observer to more precisely determine specific geometric and anatomical features for plant characterization.

## Conclusion

In this paper we proposed augmenting point data based on a new eccentricity function of the data points to improve the solution to the ellipse fitting problem. The method is somewhat analogous to the re-sampling method for Monte Carlo simulations as the method strategically adds data points in problematic regions of high eccentricity. Significant improvement was found by incorporating the procedure as a pre-processing step in five different, well-established algorithms. A strictly theoretical study undertaken to confirm that improvement can indeed be achieved in principle, shows that data augmentation is conditional for improvement. Guided by this analysis we argue that the data augmentation method proposed here can improve ellipse fitting of a realistic data set (an example of which is included for demonstration purposes), even an incomplete set arising, say, from partial occlusion of data points, provided a sufficient number of high eccentricity points can be found at one end of the major axis.

The concept we have introduced of data point eccentricity and the subsequent data augmentation procedure we have proposed can be adapted naturally to other fitting problems where there is provision for assigning non-uniform weights to data points and then re-sampling according to these weights. The results suggest that the proposed method would likely result in improved quality of fit when integrated within other ellipse fitting algorithms.

The method has here been applied to an ellipse fitting problem involving a realistic data set arising in a root phenotyping exercise where occlusion of data points is an inherent possibility. The procedure was shown to result in significant improvement in the quality of 3D reconstruction of the root system architecture. We anticipate that the method could be integrated quite readily in multiple ellipse fitting procedures [40], which we aim to demonstrate in a future publication which explores this possibility and its applications.
